# Predictive Risk Factors for Retinal Redetachment Following Uncomplicated Pars Plana Vitrectomy for Primary Rhegmatogenous Retinal Detachment

**DOI:** 10.3390/jcm9124037

**Published:** 2020-12-14

**Authors:** Josef Guber, Maico Bentivoglio, Christophe Valmaggia, Corina Lang, Ivo Guber

**Affiliations:** 1 Eye Clinic, Cantonal Hospital Sankt Gallen, CH-9007 St. Gallen, Switzerland; maico.bentivoglio@gmail.com (M.B.); christophe.valmaggia@kssg.ch (C.V.); corina.lang@kssg.ch (C.L.); 2 Department of Ophthalmology, University Hospital Basel, 4031 Basel, Switzerland; 3 Department of Ophthalmology, University of Geneva, 1205 Geneva, Switzerland; ivo_guber@hotmail.com

**Keywords:** cryocoagulation, endolaser, phacoemulsification, rhegmatogenous retinal detachment, risk factors, vitrectomy

## Abstract

Purpose: To investigate clinical and surgical factors influencing the outcome after primary rhegmatogenous retinal detachment surgery. Methods: A retrospective, single-centre, case-control study of 1017 eyes of 1017 consecutive patients with primary rhegmatogenous retinal detachment (RRD) who underwent pars plana vitrectomy (PPV), were included in the study. Analysed surgical factors were: combined procedure with phacoemulsification, type of retinopexy (cryocoagulation, endolaser, combined), type of tamponade (gas, silicone oil), and anatomical factors: primary proliferative vitreoretinopathy (PVR) and macular detachment at the time of surgery. Results: Overall retinal re-detachment rate was 10.1%. The main reason for re-detachment was an insufficient retinopexy in 53.6%, followed by PVR (37.3%), and retinal detachment occurred at a different location caused by another break in 9.1%. No significant difference in the rate of re-detachment was found if a phacoemulsification with simultaneous IOL implantation was performed (*p* = 0.641). No significant difference between the various retinopexy techniques was found (*p* = 0.309). Risk factors re-detachment were primary PVR (*p* = 0.0003), silicone oil as initial tamponade (*p* = 0.0001) as well as macula off detachments (*p* = 0.034). Conclusions: The present study showed no significant difference between the types of retinopexy and if additional phacoemulsification was performed or not. Factors associated with a higher risk for re-detachment were detached macula at surgery, primary PVR and primary oil-filling.

## 1. Introduction

In 1972 Robert Machemer revolutionised the treatment of rhegmatogenous retinal detachment (RRD) by introducing pars plana vitrectomy (PPV) as an alternative to scleral buckling [1]. Since then, technological advancements in PPV have led to the introduction of small-incision PPV, and it has become the most common procedure used to treat RRD, while scleral buckling is used only for select cases [2,3,4,5].

A major disadvantage of PPV is the development and progression of cataracts, which occur in up to 80% of the phakic patients within one postoperative year [6,7]. In addition, the use of intraocular gas—or silicone oil tamponade contributes to the progression of cataracts [8,9]. Therefore, combined procedures have been increasingly used in the last few years [10,11,12,13].

Previous studies have published success rates of PPV for the repair of primary RRD, ranging from 82.4% to 93.5% [2,14,15,16]. Known risk factors include proliferative vitreoretinopathy (PVR) and giant retinal tears, which are associated with a higher incidence of redetachment [17]. However, several other surgical and clinical factors, which have not been discussed to date could influence the success rate of the surgical repair of RRD, such as the addition of phacoemulsification simultaneously or type of retinal retinopexy. The retinopexy is an integral part of every procedure for retinal detachment repair [18,19]. Cryocoagulation and endolaser lead to scarring and a durable chorioretinal adhesion within 1–3 postoperative weeks [20,21]. To date, the choice of retinopexy has been based on the preference of the vitreoretinal surgeon, and there have been no studies comparing both the two techniques. However, some previous studies have shown that cryotherapy can be a stimulating factor for PVR and is, therefore, associated with a higher risk for surgical failure [22].

The aim of this study was to analyse various clinical and surgical factors affecting the failure rate of PPV for the repair of primary RRD in terms of retinal re-detachment as well as a review of the current literature.

## 2. Material and Methods

This retrospective, single-centre, case-control study included 1017 eyes of 1017 patients with primary RRD who underwent PPV. The study was approved by the local ethics committee EKOS (BASEC Nr. 2018-00104) and adhered to the tenets of the Declaration of Helsinki.

We included the patients with primary RRD, who underwent PPV at Cantonal Hospital St. Gallen (tertiary referral centre) in the period between January 2013 and December 2017. Patients with tractional or exudative retinal detachment were excluded. Collected data included demographics, eye involved, macular status, presence of PVR of any stage at the first preoperative visit, lens status (phakic or pseudophakic), type of tamponade (silicone oil or intraocular gas), occurrence of redetachment, and cause of redetachment (PVR, insufficient retinopexy, or new detachment).

The surgeries were performed by three experienced vitreoretinal surgeons within 24 h. The type of retinopexy was solely chosen based on the surgeon’s preference during the procedure. In phakic patients, phacoemulsification and PPV were performed simultaneously by the same surgeon.

In accordance with the hospital policy, patients were reviewed by the operating surgeon at our outpatient clinic for the first three months. Further follow-ups were conducted by a private ophthalmologist. In cases of redetachment after the initial three months, patients were referred to the hospital again for reoperation.

### 2.1. Surgical Technique

A standard 23-gauge PPV (Stellaris, Bausch + Lomb, New York, NY, USA) was performed under general anaesthesia. After core and complete peripheral PPV, retina reattachment was achieved with fluid-air exchange and drainage of the subretinal fluid via the retinal tear or with the use of heavy liquid followed by fluid-air exchange. If required, retinotomies were performed to achieve thorough retinal reattachment. Cryocoagulation or endolaser was used for retinopexy. The type of retinopexy was selected based on the surgeon’s preference. In some eyes, such as those with a large lattice area or multiple breaks, both types of retinopexy were used. Internal limiting membrane (ILM) peeling of the posterior pole was not performed in any patient. Finally, gas (20% sulphur hexafluoride, 12% perfluoropropane) or silicone oil (silicone oil 5000 cs, Densiron oil) was used as intraocular tamponade. The selection of tamponade depended on the aspects of RRD, e.g., 20% sulphur hexafluoride was used if retinal breaks were present in the upper quadrants while 12% perfluoropropane gas was used when retinal tears were located inferiorly and in patients with difficulties in posturing. The use of silicone oil tamponade was reserved for complex cases, such as in the presence of PVR or in RRDs with giant retinal tears. In all phakic eyes, phacoemulsification with intraocular lens implantation in the bag combined with PPV was performed simultaneously.

### 2.2. Statistical Analysis

The binary outcome was whether retinal detachment re-occurred or not. The investigated predictors were additional phacoemulsification, the type of retinopexy, primary PVR, the type of tamponade, and macular status. Because the rate of redetachment depends on the presence of PVR and type of tamponade (e.g., primary silicone oil in giant retinal tears), we formed subgroups (eyes with primary PVR or primary silicone oil tamponade) to prevent these factors from confounding the predictor-outcome associations. Furthermore, we performed adjusted analyses to account for clinical characteristics (macular status). A chi-squared test with continuity correction was used to analyse the predictor-outcome association for all patients the high-risk subgroups.

We used generalised linear models with binomial error distribution to test whether predictor-outcome associations depended on PVR or the type of tamponade or not and for adjusted analyses considering clinical characteristics (macular status).

For demonstrating that the study was adequately powered to detect a significant and expected difference in the redetachment rates, our clinical trial unit performed a power calculation. In this calculation, the number of patients in each group required to show a significant difference of 90% was 342. All our analyses were performed with R, v 3.3.3.

## 3. Results

A total of 1017 consecutive eyes of 1017 patients were included in the present study. The age ranged from 15.2 to 94.5 years, and the median age was 63.2 years. The macula detached in 587 patients (57.7%). PVR was present preoperatively in 93 patients (9.1%). In 138 patients (13.6%), silicone oil was used as the primary tamponade after vitrectomy. Table 1 shows the patient characteristics in detail.

Overall, in 103 patients (10.1%), the retina detached again. However, if PVR was present, the failure rate was double (21.5%). The most common cause for redetachment was an insufficiently treated break, which occurred in 53.6% of cases, followed by postoperative PVR-reaction, which occurred in 37.3%. In 9.1% of cases, another break at a different location redetached the retina.

No significant differences in the rate of redetachment were found in the failure rate of PPV with and without additional phacoemulsification performed simultaneously (*p* = 0.641) or among different types of retinopexy used (*p* = 0.309). Patients with a macula-off detachment showed a significantly higher redetachment rate (*p* = 0.034; Table 1). 

Further analysis of the type of retinopexy showed that cryotherapy is not associated with a higher rate of redetachment caused by PVR compared to endolaser (*p* = 0.47; Table 2). 

The subgroup analysis revealed a significantly higher rate of redetachment in eyes with primary PVR or silicone oil as the primary tamponade (*p* = 0,0003 and *p* = 0.0001, respectively). There was a strong association between these two confounders (X^2^ = 290, *p* < 0.0001). Nevertheless, when associations between additional phacoemulsification, type of retinopexy and retinal redetachment were analysed separately for these high-risk subgroups, the result was not different from those for all patients (Table 3).

## 4. Discussion

In the last few years, PPV has become the preferred technique for the treatment of RRD [2,3,4]. However, a disadvantage of PPV is the postoperative development of cataracts [6,7]. Therefore, many vitreoretinal surgeons have begun to perform PPV combined with phacoemulsification. The removal of cataract before performing vitrectomy optimises visualisation in the posterior pole and periphery and allows for good access to the vitreous base without the risk of touching the lens. Thus, it is possible to perform a more extensive shaving of the periphery with a more-complete vitrectomy. In addition, combined surgery may provoke a more intense postoperative inflammation with a higher rate of posterior synechiae formation [23,24]. Some lens-related complications have been reported with the use of intraocular tamponade, such as pupillary capture, lens decentration, and anterior displacement of the lens, which may induce a myopic shift [25,26]. With our study, we were able to prove that a combined procedure can safely be performed without worsening the impact on the postoperative outcome in terms of retinal redetachment [27]. However, a new localised vitrectomy technique for phakic patients with uncomplicated RRD introduced by Bonfiglio et al. has shown a high anatomical success rate without causing progression of cataract [28].

Creating a sufficient barrier around retinal breaks is another crucial step in the treatment of RRD [18,19,20]. Retinopexy is usually performed using cryocoagulation or endolaser. Both types of retinopexy cause an alteration in the blood–retinal barrier, which leads to a breakdown of this barrier and frees retinal pigment epithelium cells, which can lead to PVR [29]. However, in our study population, neither retinopexy type showed a higher risk for failure [30].

PVR is an important factor for success after retinal detachment surgery because of its association with retinal traction and detachment caused by proliferative cells with contractive ability. In previous studies, the incidence of primary PVR before surgery was reported in 5–11% of the eyes with RRD [3,17,31]. Furthermore, in 50–75% cases, PVR is a cause of redetachment after the surgery [31].

In our study, PVR was a cause of redetachment in 37.3% of cases. The leading cause for surgical failure was insufficient retinopexy, which occurred in 53.6% of cases. Retinal redetachment caused by another break at a different location was seen in 9.1% of cases.

Further analysis of the different retinopexy methods showed that using cryocoagulation was not associated with a higher degree of redetachments caused by PVR. However, although not statistically significant, cryocoagulation had a slightly higher rate for insufficiency of the retinopexy and redetachments at a different location caused by another break (Table 2). In our experience, most redetachments caused by insufficient retinopexy are caused by overtreatment with cryocoagulation causing an atrophic scar around the retinal break [30].

After performing retinopexy of either type, there is an initial reduction in the adhesive strength during the first week, which is caused by cellular infiltration in this area or an oedema, and a sufficient adhesion is usually reached after 2–3 weeks [20,21]. To overcome this time span, intraocular tamponade is required. The tamponade displaces the subretinal and preretinal fluid and apposes the retina to the underlying pigment epithelium [32,33]. The type of tamponade is select based on the degree of severity of the retinal detachment. Silicone oil tamponade is usually used in more complex retinal detachment, e.g., in PVR detachments or giant retinal tears, which explains the higher redetachment rate in this group (19.6%).

Recently, a new type of retinopexy has shown promising results with the creation of an immediate chorioretinal adhesion using high-frequency electric welding [20]. Because of this immediate chorioretinal adhesion, the use of tamponade could be unnecessary.

Furthermore, the eyes with a detached macula at the time of surgery also have a higher risk for surgical failure. This could probably be because of the longer duration and greater extent of retinal detachment [34].

## 5. Conclusions

This study shows that there are no significant differences in the redetachment rate after PPV with and without additional phacoemulsification performed simultaneously. Furthermore, the type of retinopexy had no impact on the failure rate, and cryotherapy was not associated with higher rates of surgical failure caused by PVR. The risk factors that were associated with a higher rate for redetachment were primary PVR and the use of silicone oil as tamponade. However, there was a strong association between these two confounders. Furthermore, extensive retinal detachments with macula off are associated with a significantly higher re-detachment rate.

## Figures and Tables

**Table 1:** Patients’ characteristics and associations between the occurrence of re-detachment and type of retinopexy, risk factors (primary PVR, silicon oil) and patient’s characteristics (macula status).

Predictor.		Re-Detachment			Chi-Squared Test	
		No (*n*)	Yes (*n*)	Yes (%)	X^2^	*p*-Value
Surgical factors	Phacoemulsification				0.22	0.641
	No	461	55	10.7		
	Yes	453	48	9.6		
	Retinopexy					
	Cryocoagulation	435	57	11.6	2.35	0.309
	Endolaser	201	18	8.2		
	Combined	278	28	9.2		
	Tamponade					
	Gas	803	76	8.6	14.45	0.0001
	Silicone oil	111	27	19.6		
Anatomical factors	PVR					
	No	841	83	9.0	13.21	0.0003
	Yes	73	20	21.5		
	Macula status					
	On	397	33	7.7	4.47	0.034
	Off	517	70	11.9		
Patient characteristics	Sex					
	Female	318	37	10.4	0.01	0.905
	Male	596	66	10.0		
	Side					
	Left	505	47	8.5	3.08	0.079
	Right	409	56	12.0		
	Age (years)				t = −1.1	0.273
	Median	63	68			

**Table 2:** Significance of association between retinopexy methods and cause of re-detachment.

Retinopexy	Cause of Re-Detachment						Chi-Squared Test	
	PVR		Insufficiency		New Break			
	*n*	%	*n*	%	*n*	%	X^2^	*p*-Value
Endolaser	11	27	8	13	1	10	3.59	0.47
Cryo	19	46	35	59	6	60		

**Table 3:** Association between the occurrence or re-detachment and type of retinopexy for high-risk subgroups (with primary PVR or silicon oil tamponade).

	Re-Detachment			Chi-Squared Test	
	No (*n*)	Yes (*n*)	Yes (%)	X^2^	*p*-Value
**Primary PVR (*n* = 93)**					
Phaco					
No	47	10	17.5	0.83	0.362
Yes	26	10	27.8		
Retinopexy					
Cryocoagulation	27	8	22.9	0.59	0.744
Endolaser	32	7	18.0		
Combined	14	5	26.3		
**Primary silicone oil (*n* = 138)**					
Phaco					
No	66	14	17.5	0.25	0.617
Yes	45	13	22.4		
Retinopexy					
Cryocoagulation	49	10	17.0	0.47	0.791
Endolaser	38	11	22.0		
Combined	23	6	20.7		

## Data Availability

The datasets used and/or analysed during the current study are available from the corresponding author on reasonable request.
